# Impact of temperature, humidity, dehydration, and psychological stress on salivary flow and xerostomia in young men: An observational study

**DOI:** 10.1371/journal.pone.0349221

**Published:** 2026-05-12

**Authors:** Kayoko Ito, Kaname Nohno, Saori Funayama, Kouji Katsura, Noboru Kaneko, Keiko Tanaka, Makoto Inoue

**Affiliations:** 1 Oral Rehabilitation, Niigata University Medical and Dental Hospital, Niigata, Japan; 2 Division of Oral Science for Health Promotion, Niigata University Graduate School of Medicine, Dentistry and Health Sciences, Niigata, Japan; 3 Department of Oral Radiology, Niigata University Medical and Dental Hospital, Niigata, Japan; 4 Division of Preventive Dentistry, Niigata University Graduate School of Medicine, Dentistry and Health Sciences, Niigata, Japan; 5 Department of Patient Synthesis Support Center, Niigata University Medical & Dental Hospital, Niigata, Japan; 6 Division of Dysphagia Rehabilitation, Niigata University Graduate School of Medicine, Dentistry and Health Sciences, Niigata, Japan; Tribhuvan University, NEPAL

## Abstract

Xerostomia, or subjective oral dryness, is commonly reported and may occur with or without objectively reduced salivary secretion (hyposalivation). To assess salivary gland function, the salivary flow rate must be repeatedly measured over time under consistent conditions. However, given that temperature and humidity vary throughout the year, standardized measurement is difficult. Some reports have indicated that the unstimulated salivary flow rate is lower in summer than in winter, although no studies have evaluated this pattern in conjunction with humidity or dehydration. This longitudinal observational study aimed to determine the extent to which room temperature, relative humidity, body dehydration, and psychological stress are associated with unstimulated and stimulated salivary flow rates and xerostomia in healthy young men (n = 39). A questionnaire was administered to collect data regarding participants’ body mass index, medical history, medications, and degree of xerostomia (severe, moderate, mild, or none). Unstimulated and stimulated salivary flow rates, salivary cortisol and chromogranin A concentrations, urine specific gravity, room temperature, and relative humidity were measured. Psychological stress was assessed via measurements of salivary cortisol and chromogranin A concentrations. Urine specific gravity served as an indicator of hydration status. Factors influencing the salivary flow rate and xerostomia were analyzed using generalized estimating equations. The unstimulated salivary flow rate was significantly associated with temperature (p = 0.008), humidity (p = 0.039), urine specific gravity (p = 0.045), and chromogranin A concentration (p < 0.001); the stimulated salivary flow rate was not significantly linked to these factors. Furthermore, xerostomia was only related to chromogranin A concentration (p = 0.012). These results suggest that indoor environmental conditions and hydration status should be considered when interpreting changes in unstimulated salivary flow rate over time; subjective oral dryness (xerostomia) showed a significant association only with chromogranin A in this exploratory study.

## Introduction

Xerostomia, or subjective oral dryness, is common in older adults. The estimated prevalence of xerostomia is 37.8% in independent older adult populations [1] and 63% in hospitalized patients [2]. Xerostomia can also occur in younger individuals; Mizutani et al. reported an incidence of 8.8% among 2077 students aged 18–24 years [3]. Xerostomia often accompanies hyposalivation (objectively reduced salivary secretion). Major contributors to hyposalivation include adverse medication effects, Sjögren’s syndrome, psychological conditions, and irradiation [4,5]. Reduced salivary secretion can substantially diminish quality of life and is associated with eating, swallowing, and speech difficulties; halitosis; burning sensations; altered taste perception; and increased risks of caries, gingivitis, periodontal disease, candidiasis, and other oral infections [6].

To assess hyposalivation, subjective oral dryness, and potential treatment effects over time, patient-reported symptoms and salivary flow rates are often measured repeatedly under conditions that are as consistent as possible. Some factors that influence salivary secretion—such as posture, exposure to light [7], and time of measurement—can be standardized. However, medications and systemic conditions may change over time; they must be monitored when repeated measurements are performed. Environmental conditions such as temperature and humidity vary across seasons, and clinical factors such as hydration status and stress-system activity may fluctuate.

Seasonal variation in salivary flow has been reported. Elishoov et al. [8] reported higher salivary volumes from major salivary glands in winter than in summer. However, because their study included women, it is possible that the menstrual cycle affected salivary components [9]. Shannon [10] evaluated 3868 men aged 17–22 years in Texas, revealing higher parotid salivary flow in winter than in summer; the authors proposed dehydration as a potential explanation, but they did not measure hydration-related variables or humidity. Thus, although seasonal differences have been described, the extent to which temperature, humidity, and hydration status contribute to changes in salivary flow remains unclear.

Stimulated salivary flow is strongly influenced by mastication and taste. One study showed no seasonal variation in stimulated salivary flow [11]; however, that analysis focused on temperature alone. Investigations that consider both temperature and humidity are warranted.

Importantly, xerostomia and hyposalivation are related but distinct concepts. Xerostomia is a subjective symptom assessed by patient reporting, whereas hyposalivation refers to objectively reduced salivary flow [12]. Xerostomia does not necessarily correspond to salivary volume; it may also reflect qualitative changes in salivary composition and altered perception mechanisms [13]. To date, no studies have examined relationships between subjective oral dryness and environmental conditions (temperature and humidity) or hydration status.

When salivary flow and subjective dryness are compared over time or seasons, the magnitude of potential influences from the above factors should be considered to avoid misattributing seasonal variation to treatment effects.

The present study aimed to explore factors associated with unstimulated and stimulated salivary flow rates, as well as subjective oral dryness, in healthy young men. Because salivary secretion is influenced by systemic disease and xerogenic medications, a relatively homogeneous healthy cohort was selected to reduce confounding and establish baseline physiological associations under standardized indoor conditions in a repeated-measures design. Specifically, the associations of indoor room temperature, relative humidity, hydration status, and stress-related salivary biomarkers with salivary flow and oral dryness were examined to improve the interpretation of longitudinal measurements.

## Materials and methods

### Participants

This study was conducted in accordance with the principles of the Declaration of Helsinki. The Ethics Committee of the Niigata University Graduate School of Medical and Dental Sciences approved the protocol (2017−0260), and all participants provided written informed consent. In this study, the same group of participants was repeatedly evaluated across four seasons.

To reduce potential variability from systemic illness and medication use, the study cohort comprised healthy young men who were students at Niigata University of Health and Welfare. Recruitment occurred from June 18 to June 23, 2018. Women were excluded from the study to reduce potential variability from hormonal influences such as menstruation. Participants were excluded if they had any known disease or condition that could affect salivary secretion or if they had used medications known to affect salivary secretion within the 2 weeks prior to the start of measurements. Iron supplementation was not used as an exclusion criterion because its mechanism of action is not thought to reduce salivary secretion. Accordingly, participants taking a medication were eligible only if it was not expected to affect salivary secretion. The sample size was calculated using G*Power with an effect size of 0.35, an α value of 0.05, and a β value of 0.2, based on a previous study [14]. The required sample size was 41 participants after adjustment for potential dropouts.

### Measurements

Measurements were performed at four time points (one measurement per time point): summer (July 2018), autumn (October 2018), winter (January 2019), and spring (April 2019) in Japan. Two or three measurement days were allocated for each season. An air conditioner was used to maintain a constant room temperature during the measurement periods. To minimize acute effects, participants were instructed to refrain from eating, drinking, brushing their teeth, or engaging in strenuous physical activity (e.g., running or exercise) for at least 1 hour before each measurement; however, habitual physical activity was not otherwise standardized or objectively recorded. All measurements were performed between 9:00 am and 12:00 pm to reduce the impact of circadian variation, and each participant was assessed at the same time of day across the four seasonal measurement sessions. Medical history and medication use were recorded by questionnaire (as indicated in the next section).

### Measurement items

Factors that could be controlled, such as room brightness and participant posture [7], were kept as stable as possible. The following variables were collected at each session: indoor room temperature and relative humidity; unstimulated and stimulated salivary flow rates; salivary cortisol and chromogranin A (CgA) concentrations; urine specific gravity; and questionnaire-based measures, including subjective oral dryness, medical history, medication use, height, and weight. Room temperature and humidity at the time of measurement were recorded using a digital thermometer/hygrometer (Tanita, Japan). Participants completed a questionnaire that collected information regarding height, weight, medical history, and medication use. They reported their subjective degree of oral dryness on the following scale: severe, moderate, mild, or none. This four-level oral dryness scale was selected to minimize participant burden in repeated seasonal measurements and to maintain comparability with prior observational questionnaire studies that used the same response options [15,16]. However, the exploratory scale has not been formally validated as a xerostomia instrument and is not intended to be directly comparable to established multi-item xerostomia instruments.

After entering the measurement room, participants were seated for at least 10 minutes to acclimate to the stable indoor conditions before measurements were taken. All salivary flow and xerostomia assessments were conducted under these controlled indoor conditions; the environmental variables used for analysis were the indoor room temperature and relative humidity recorded at the time of saliva collection. However, the duration of time spent indoors before entering the measurement room and the extent of recent outdoor exposure were not recorded; therefore, the present protocol cannot distinguish the effects of measured indoor conditions from potential residual effects of outdoor exposure. All objective measurements were performed by a dentist. The unstimulated salivary flow rate was measured using the spitting method, in which participants spat into a cup for 5 minutes. Although this approach is commonly used to quantify unstimulated whole saliva, it requires periodic expectoration and thus may not reflect a completely motionless resting condition. To minimize potential effects of orofacial muscle activity, participants were instructed to remain relaxed and to minimize movement during the collection period, and the procedure was standardized across all sessions. Next, the stimulated salivary flow rate was measured after participants freely chewed paraffin gum (GC Corporation, Japan) for 5 minutes. The saliva was weighed, and 1 g of saliva was considered equivalent to 1 mL [17]. After collection, saliva samples were immediately frozen and stored at −80°C until biomarker analysis.

Psychological stress–related activity was assessed via the following biomarkers in frozen unstimulated saliva [18]: salivary cortisol concentrations, measured using the YK241 Cortisol (Saliva) EIA Kit (Yanaihara Institute Inc., Japan), and CgA, measured using the YK070 Human Chromogranin A EIA Kit (Yanaihara Institute Inc., Japan). Cortisol reflects hypothalamic–pituitary–adrenal axis activity, whereas salivary CgA has been reported to respond rapidly to psychosomatic stressors; therefore, these biomarkers were treated as complementary indicators rather than interchangeable measures of contemporaneous perceived stress. Associations between salivary cortisol and CgA levels and oral dryness/reduced salivary flow have been reported previously [19]. Notably, cortisol and CgA assays were performed in a single batch after completion of all four seasonal sampling sessions; therefore, the duration of continuous storage at −80°C varied by season (approximately 3–15 months).

After saliva measurements, urine was collected. Dehydration was operationally defined using urine specific gravity, measured with a clinical refractometer (MASTER-SUR/Jα; Atago Co. Ltd., Japan). This parameter has been validated in prior studies as a reliable indicator of hydration status [20].

No validated questionnaire assessing perceived stress (e.g., perceived stress scale–type instruments) was administered at the time of sampling. Additionally, potential contextual stressors related to student life (e.g., examinations) were not recorded in relation to the measurement dates.

### Statistical analysis

Body mass index was calculated as weight (kg) divided by height squared (m^2^) using self-reported height and weight obtained from the questionnaire. Descriptive statistics were used to present each parameter in each season. Continuous variables (body mass index, room temperature, humidity, salivary volume, salivary cortisol, CgA, and urine specific gravity) were summarized as means with standard deviations; the categorical variable (oral dryness) was summarized using count and percentage values. Because the study involved repeated measurements of the same participants under different seasonal conditions, this study utilized generalized estimating equations to analyze associations between environmental variables (indoor room temperature and indoor relative humidity recorded at the time of sampling) and outcomes (unstimulated salivary flow rate, stimulated salivary flow rate, degree of xerostomia). The generalized estimating equations method was selected because it is appropriate for longitudinal data with repeated measurements from the same individuals; it allows estimation of both the presence and magnitude of associations while adjusting for within-participant correlations [21]. Predictors were defined a priori as environmental (indoor room temperature and indoor relative humidity) and physiological (urine specific gravity as an index of hydration status and salivary biomarkers as indicators of stress-system activity); outdoor temperature and humidity were not included in the analyses. The primary models were exploratory, and simultaneously included the environmental and physiological variables of interest (room temperature, humidity, urine specific gravity, and a stress-related biomarker); however, additional covariates (e.g., body mass index and other potential confounders) were not included for comprehensive multivariable adjustment. Because salivary cortisol and CgA are related biomarkers of stress-system activity, they were not simultaneously included in the same generalized estimating equation models to avoid multicollinearity and unstable coefficient estimates in this dataset. Accordingly, stress-system activity was represented by a single biomarker per model; tables in the Results section present data from models that included CgA as the stress-related biomarker. An exchangeable correlation structure was assumed for repeated measures per participant. This approach was preferred over repeated-measures analysis of variance or mean regression because it does not require the assumption of normally distributed outcomes and is more robust for modeling skewed data (e.g., salivary flow rates). Stratification into comparison groups (e.g., “high” vs. “low” temperature) was not performed, given that some seasonal measurements yielded overlapping environmental values (e.g., overlapping temperature ranges in winter and spring). Xerostomia was assessed using a single, non-validated four-level scale (severe, moderate, mild, or none). For analysis, the response was dichotomized as “presence of oral dryness” (severe/moderate/mild) versus “no oral dryness” (none) as an exploratory operational definition; this approach was used to improve stability and interpretability, given the limited sample size and the ordinal nature of the four-level scale. Importantly, because the four response levels were not based on a validated xerostomia instrument and the ordinal spacing between categories is uncertain, ordinal modeling was not pursued in this exploratory analysis. All analyses were performed using SPSS version 28.0 (IBM Corp., Japan). The statistical significance threshold was set at p < 0.05.

## Results

### Participant characteristics

Of the 41 participants, 39 completed measurements in all four sessions. One participant dropped out because of discomfort caused by the paraffin gum used for the stimulated saliva measurement, and one participant was unable to undergo winter measurements because of a cold; all data regarding these participants were excluded from the analysis. The mean (± standard deviation) age and body mass index at the first measurement were 18.4 (± 0.5) years and 21.6 (± 2.7) kg/m^2^, respectively.

Three participants had asthma, and 16 participants had pollen allergies; however, they had no symptoms at the time of measurement and reported no use of allergy-related medications (including antihistamines) during the 2 weeks before each measurement session. With respect to other medications, one participant was taking an iron supplement throughout all four measurement sessions. Consistent with the eligibility criteria, no participants reported using medications known to affect salivary secretion within the 2 weeks prior to the start of measurements.

### Measurement values in each season

Table 1 presents the room temperature, humidity, unstimulated salivary flow rate, stimulated salivary flow rate, degree of xerostomia, urine specific gravity, cortisol concentration, and CgA concentration at the four measurement sessions. The temperature was highest in July (summer), and humidity was highest in October (autumn). The number of participants with xerostomia was the same in July (summer) and October (autumn) (24 participants, 60.0%) but was lower in April (spring) (18 participants, 45.0%).

**Table 1:** Measurement values in each season.

		Summer	Autumn	Winter	Spring
Temperature (°C)	Mean ± SD	28.30 ± 0.00	23.78 ± 0.91	15.22 ± 0.52	17.92 ± 0.61
Humidity (%)	Mean ± SD	52.26 ± 2.12	66.95 ± 1.62	46.46 ± 1.41	45.05 ± 2.03
Unstimulated saliva (mL/min)	Mean ± SD	0.32 ± 0.23	0.30 ± 0.20	0.39 ± 0.22	0.40 ± 0.20
Stimulated saliva (mL/min)	Mean ± SD	1.88 ± 0.78	1.78 ± 0.72	1.97 ± 0.67	1.92 ± 0.70
Xerostomia	Number (%)	24 (60.0)	24 (60.0)	23 (59.0)	18 (45.0)
Urine specific gravity	Mean ± SD	1.023 ± 0.006	1.023 ± 0.006	1.023 ± 0.006	1.024 ± 0.006
Cortisol (μg/dL)	Mean ± SD	0.25 ± 0.11	0.30 ± 0.19	0.29 ± 0.14	0.36 ± 0.20
CgA (pmol/mL)	Mean ± SD	8.26 ± 9.00	13.86 ± 21.81	10.54 ± 11.94	11.85 ± 16.17

SD: standard deviation.

CgA: chromogranin A.

Note: No statistical analyses were performed concerning values in this table.

### Factors influencing the unstimulated salivary flow rate

The results of the generalized estimating equations are shown in Table 2. Significant predictors of the unstimulated salivary flow rate were temperature, humidity, urine specific gravity, and CgA concentration. Each 1°C increase in temperature was associated with an average reduction of 0.006 mL/min in unstimulated salivary flow rate; each 1% increase in humidity was linked to a 0.003 mL/min decrease in flow rate. Furthermore, each 1-unit increase in urine specific gravity was associated with an average reduction of 4.083 mL/min in unstimulated salivary flow rate, and each 1 pmol/mL increase in salivary CgA concentration was linked to a 0.004 mL/min decrease in flow rate.

**Table 2:** Results of unadjusted generalized estimating equations used to identify factors influencing the unstimulated salivary flow rate.

	β-coefficient	Standard error	95% Confidence interval		
			Lower	Upper	P value
Intercept	4.839	2.107	0.710	8.968	0.022
Temperature	−0.006	0.002	−0.010	−0.002	0.008
Humidity	−0.003	0.001	−0.005	0.000	0.039
Urine specific gravity	−4.083	2.041	−8.083	−0.083	0.045
CgA	−0.004	0.001	−0.006	−0.002	<.001

CgA: chromogranin A.

Note: Cortisol and CgA were not entered simultaneously because of potential multicollinearity; the model shown includes CgA as the stress biomarker. The statistical significance threshold was set at P < 0.05.

### Factors influencing the stimulated salivary flow rate

The results of the generalized estimating equations are shown in Table 3. None of the assessed factors were significant predictors of the stimulated salivary flow rate.

**Table 3:** Results of unadjusted generalized estimating equations used to identify factors influencing the stimulated salivary flow rate.

	β-coefficient	Standard error	95% Confidence interval		
			Lower	Upper	P value
Intercept	−4.866	6.339	−17.290	7.557	0.443
Temperature	−0.010	0.010	−0.030	0.010	0.320
Humidity	−0.005	0.003	−0.011	0.001	0.127
Urine specific gravity	7.038	6.213	−5.138	19.214	0.257
CgA	0.002	0.003	−0.004	0.008	0.532

CgA: chromogranin A.

Note: Cortisol and CgA were not entered simultaneously because of potential multicollinearity; the model shown includes CgA as the stress biomarker. The statistical significance threshold was set at P < 0.05.

### Factors influencing the degree of xerostomia

The results of the generalized estimating equations are shown in Table 4. When xerostomia was analyzed as a binary outcome (presence vs. absence of oral dryness), the only factor significantly associated with xerostomia was the CgA concentration: each 1 pmol/mL increase in salivary CgA concentration was associated with a 0.019-unit decrease in the log-odds of reporting xerostomia. Room temperature, humidity, and urine specific gravity were not significantly linked to xerostomia.

**Table 4:** Results of unadjusted generalized estimating equations used to identify factors influencing the degree of xerostomia.

	β-coefficient	Standard error	95% Confidence interval		
			Lower	Upper	P value
Intercept	−48.146	32.317	−111.486	15.194	0.136
Temperature	0.013	0.030	−0.046	0.072	0.666
Humidity	0.022	0.018	−0.013	0.057	0.222
Urine specific gravity	46.120	31.564	−15.743	107.984	0.144
CgA	−0.019	0.008	−0.033	−0.004	0.012

CgA: chromogranin A.

Note: Cortisol and CgA were not entered simultaneously because of potential multicollinearity; the model shown includes CgA as the stress biomarker. The statistical significance threshold was set at P < 0.05.

## Discussion

To the authors’ knowledge, this is the first study to use generalized estimating equations to examine the associations of room temperature, humidity, psychological stress, and dehydration with the unstimulated salivary flow rate, stimulated salivary flow rate, and degree of xerostomia in the same participants over time. The results suggest that the unstimulated salivary flow rate was associated with temperature, humidity, psychological stress, and dehydration. For example, a 10-degree increase in temperature was associated with a 0.06 mL/min decrease in the unstimulated salivary flow rate, whereas a 10% increase in humidity was associated with a 0.03 mL/min decrease. The average unstimulated salivary flow rate for healthy young men is 0.35 mL/min [22]. Therefore, a change of 0.06 mL/min in the unstimulated salivary flow rate with a 10-degree temperature increase corresponds to an approximate change of 17%. Accordingly, variation in indoor environmental conditions may contribute to within-person differences in unstimulated salivary flow over time in healthy individuals, and these factors should be considered when interpreting longitudinal measurements in research settings.

The salivary flow rate reportedly varies throughout the year; the unstimulated salivary flow rate is greater in winter than in summer [8]. Shannon [10] evaluated male military personnel in Texas and found that the salivary flow rate was greater in winter than in summer; however, repeated measurements of the same participants were not performed. Kavanagh et al. [14] measured the salivary flow rate of 12- to 13-year-old boys in North Wales; they found that flow rates were lowest in September and highest in February, demonstrating an inverse association between salivary flow and ambient temperature. These findings are consistent with the present results. A study of 26 men and 24 women in Sri Lanka also showed that the unstimulated salivary flow rate decreased as the temperature increased [23]. The authors discussed the effect of temperature on salivary flow rate using a sine wave. One method for assessing seasonal variation involves comparison of salivary flow rates across the four seasons using analysis of variance. Although measurements were taken in all four seasons, temperatures in the present study were 28.30°C in summer, 23.78°C in autumn, 15.22°C in winter, and 17.92°C in spring; thus, winter and spring temperatures were relatively close and showed partial overlap compared with summer and autumn. Furthermore, in addition to repeated measurements of the same participants, the aim of the present study was to simultaneously examine multiple factors (temperature, humidity, and urine specific gravity). Therefore, generalized estimating equations were utilized. The results showed that the salivary flow rate decreased as humidity increased. Notably, this study did not examine whether participants were mouth-breathing; however, low humidity increases the evaporation of saliva, which may explain why humidity was related to the unstimulated salivary flow rate.

Indicators of dehydration include plasma osmolality, saliva osmolality, urine color, and urine specific gravity. Although saliva osmolality can be used as an indicator when dehydration reaches 3% [24], it cannot be used to evaluate patients with 7% dehydration because of large individual variation [25]. Furthermore, urine color, urine specific gravity, and salivary flow rate can be used to determine hydration status in young people [26] but not in older adults [27]. Therefore, urine specific gravity served as an indicator of dehydration in the present study, revealing a strong correlation between urine specific gravity and the unstimulated salivary flow rate. This finding supports the theory that low saliva volume in summer may be due to dehydration [10]. In the present study, participants were instructed not to drink water for 1 hour before measurement to avoid the effects of food and drink on salivary secretion, which may have increased short-term dehydration, particularly in summer. Accordingly, hydration status should be considered when interpreting unstimulated salivary flow measurements obtained under similar pre-measurement restrictions in research settings.

Our results also showed that the stimulated salivary flow rate was not associated with temperature, humidity, or psychological stress. Stimulated saliva is secreted in response to chewing and gustatory stimuli; these effects may be strong enough to mask the associations of the other assessed factors. In this exploratory dataset, environmental conditions and the assessed physiological variables were not significantly associated with stimulated salivary flow; confirmation in larger cohorts and clinical populations is warranted.

We hypothesized that xerostomia would be associated with humidity and temperature because humidification has been reported to alleviate symptoms of oral dryness in patients with Sjögren’s syndrome [28]. However, the present study showed that xerostomia was not associated with humidity or temperature but was instead linked to psychological stress. Kanzow et al. [29] suggested that xerostomia experienced while wearing a mask during the coronavirus disease 2019 (COVID-19) pandemic was caused by stress and anxiety, given that the degree of xerostomia increased without a decrease in unstimulated salivary secretion. It is also possible that environmental humidity indirectly influences xerostomia by affecting airway evaporation and dehydration, but such relationships were not demonstrated in the present study. CgA is widely used as a biological marker of the stress system because it responds rapidly and sensitively to psychosomatic stressors [19]. A previous study demonstrated that CgA concentration is significantly associated with the degree of xerostomia [19]. However, objective salivary volume does not always correspond with the subjective sensation of xerostomia [30,31]. Accordingly, xerostomia was not treated as dependent on salivary flow rate; discordance between subjective dryness and measured salivary volume is both plausible and expected. In the present study, the unstimulated salivary flow rate was significantly associated with psychological stress, temperature, humidity, and dehydration; the degree of xerostomia was only linked to psychological stress. Given the non-validated symptom measure and dichotomization approach, the xerostomia-related findings should be interpreted cautiously and considered hypothesis-generating.

All participants in the present study were young men. This homogeneous cohort was selected to reduce confounding from systemic disease, xerogenic medication use, and sex-related physiological variation in an exploratory repeated-measures design. However, this decision limits generalizability, particularly because xerostomia is more prevalent in women. Future studies should include women and evaluate whether associations with environmental conditions, hydration status, and stress-related biomarkers differ by sex, including consideration of menstrual phase and menopausal status.

### Study limitations

This study has several limitations. First, the cohort comprised healthy young male students from a single institution; therefore, the findings may not be generalizable to other populations, including women, older adults, and individuals with systemic conditions. Although this homogeneous cohort improved internal validity for estimating associations with indoor environmental conditions and hydration status, future studies should extend these observations to older adults and clinical populations in whom xerostomia and hyposalivation are more prevalent.

Second, all measurements were conducted indoors; therefore, the results reflect associations under standardized indoor conditions rather than direct effects of outdoor seasonal exposure. Additionally, the duration for which participants had been indoors after being outdoors was not recorded. When large differences exist between outdoor and indoor temperatures, physiological adaptation requires an acclimation period. Ligtenberg et al. [32] used an interval of 5 minutes, whereas Elishoov et al. used 30 minutes [8]. In the present study, participants remained in the room for at least 10 minutes before measurements were performed; it remains uncertain whether this acclimation period was sufficient to eliminate temperature-related effects on salivary flow. Future studies with more controlled exposure protocols (e.g., standardized acclimation periods and/or controlled environmental settings) may better isolate temperature and humidity effects. Moreover, although generalized estimating equations enabled associations to be modeled across repeated measurements, the absence of stratified exposure groups hindered causal inference and reduced the ability to directly compare “high” and “low” exposure conditions.

Third, saliva collection and measurement procedures impose additional constraints. Within each season, saliva was collected on a single day per participant. Given that salivary secretion can vary among days, reliance on a single measurement per season may have reduced reliability. Furthermore, unstimulated whole saliva was collected using the spitting method, which requires periodic expectoration and may influence the measured flow rate; however, the procedure was standardized across all sessions. Finally, only whole salivary flow rate was measured. Because the parotid glands may be more susceptible to room temperature changes than the submandibular and sublingual glands [8], future studies should obtain more detailed information by separately measuring saliva secreted from each salivary gland.

Fourth, dehydration and stress-system activity were assessed using a single physiological marker [20] (urine specific gravity) and two salivary biomarkers [18] (cortisol and CgA). Although these measures have been validated, no validated questionnaire assessing perceived stress was administered; perceived stress at the time of sampling was not directly measured. Accordingly, cortisol and CgA should be interpreted as physiological biomarkers and may not reflect contemporaneous perceived stress. Additionally, cortisol and CgA reflect distinct stress-response pathways and may exhibit different temporal dynamics; thus, complete concordance between these biomarkers is not expected. The use of additional instruments (e.g., multi-item psychological stress inventories for subjective stress and plasma osmolality for dehydration) would enhance reproducibility.

Fifth, subjective oral dryness was assessed using a single, non-validated four-level scale. Dichotomization of responses as “presence” versus “absence” of oral dryness was an exploratory operational definition and may be considered arbitrary. This binary coding may have oversimplified subjective dryness and reduced the resolution of symptom variation. Future studies should use validated instruments for subjective symptom assessment, such as the Xerostomia Inventory [33], the Xerostomia Questionnaire [34], or visual analogue scales, to more comprehensively evaluate xerostomia.

Finally, although several potential confounders were controlled by study design, residual confounding cannot be excluded. Factors such as unrecorded dietary habits, fluctuating allergic activity, subtle circadian variation, or unmeasured lifestyle variables may have influenced salivary flow. Physical activity was controlled only through pre-measurement instructions (avoid strenuous exercise for ≥1 hour), and habitual or earlier same-day activity was not objectively measured or standardized; therefore, residual confounding by physical activity cannot be excluded. Moreover, although generalized estimating equations were utilized, comprehensive multivariable adjustment for confounders would have enhanced statistical rigor. This adjustment was not performed because the analyses were intended to be exploratory; inclusion of multiple additional covariates was not feasible given the sample size and repeated-measures structure. Future studies incorporating repeated within-season sampling, validated symptom instruments, and more comprehensive adjustment in clinically relevant populations are needed to confirm and extend our exploratory observations.

## Conclusion

The unstimulated salivary flow rate was significantly associated with measured indoor temperature, relative humidity, urine specific gravity, and a stress-related biomarker; the stimulated salivary flow rate was not significantly associated with these factors in the present study. Subjective oral dryness (xerostomia) was not significantly associated with temperature or humidity; it was significantly associated only with CgA. Overall, these findings should be interpreted as exploratory physiological observations in healthy young men assessed under standardized indoor conditions. Confirmation in older adults and clinical populations, using repeated within-season sampling, validated xerostomia instruments, and more rigorous modeling, is needed before broader conclusions can be inferred.
